# New perspectives on central and peripheral immune responses to acute traumatic brain injury

**DOI:** 10.1186/1742-2094-9-236

**Published:** 2012-10-12

**Authors:** Mahasweta Das, Subhra Mohapatra, Shyam S Mohapatra

**Affiliations:** 1 Nanomedicine Research Center, University of South Florida Morsani College of Medicine, 12901 Bruce B. Downs Blvd., Tampa, FL, 33612, USA; 2 Department of Internal Medicine, Division of Translational Medicine, University of South Florida Morsani College of Medicine, 12901 Bruce B. Downs Blvd., Tampa, FL, 33612, USA; 3 Department of Molecular Medicine, University of South Florida Morsani College of Medicine, 12901 Bruce B. Downs Blvd., Tampa, FL, 33612, USA; 4 James A. Haley Veteran’s Hospital and Medical Research Center, 13000 Bruce B. Downs Blvd., Tampa, FL, 33612, USA

**Keywords:** Traumatic brain injury, Blood–brain barrier, Neuroinflammation, Cytokines, Chemokines, Stem cells

## Abstract

Traumatic injury to the brain (TBI) results in a complex set of responses involving various symptoms and long-term consequences. TBI of any form can cause cognitive, behavioral and immunologic changes in later life, which underscores the problem of underdiagnosis of mild TBI that can cause long-term neurological deficits. TBI disrupts the blood–brain barrier (BBB) leading to infiltration of immune cells into the brain and subsequent inflammation and neurodegeneration. TBI-induced peripheral immune responses can also result in multiorgan damage. Despite worldwide research efforts, the methods of diagnosis, monitoring and treatment for TBI are still relatively ineffective. In this review, we delve into the mechanism of how TBI-induced central and peripheral immune responses affect the disease outcome and discuss recent developments in the continuing effort to combat the consequences of TBI and new ways to enhance repair of the damaged brain.

## Introduction

Traumatic brain injury (TBI) is a complex process involving a broad spectrum of symptoms and long-term consequences including disabilities. It is a serious health problem in the United States and around the world. Recent data show that approximately 1.7 million people sustain a TBI annually [1,2] including U.S. soldiers involved in combat operations and public safety personnel surviving terrorist attacks. An estimated 150 to 300,000 military personnel from Operation Iraqi Freedom and Operation Enduring Freedom suffered from TBI [3-5]. It contributes to 30% of all injury-related deaths and costs about $60 billion annually. TBI of any form, mild to severe, can cause intellectual and cognitive deficits, mood and behavioral changes both short- and long-term [6-9]. In the long term, these can cause potentially permanent changes and may lead to post-traumatic stress disorder (PTSD) in the general population as well as those in the military. Besides psychological symptoms, immune suppression from TBI and subsequent infections are important consequences [10].

Although TBI can range from mild to severe, most TBI is mild and characterized by brief changes in mental status and cognitive ability [11]. Although the consequences of mild TBI are not readily appreciated, it can still cause infrastructural damage to the brain and secondary axonal injury [12] and shows symptoms like cognitive or intellectual deficits and behavioral and personality changes even six months after injury [10]. In most patients suffering from mild brain injury, the symptoms disappear within six months but many others suffer in a variety of ways that may be underappreciated and treated inadequately or improperly. Even under asymptomatic conditions, unhealed neurodegeneration may cause a spectrum of diseases with huge cost to society [10].

**Figure 1:**
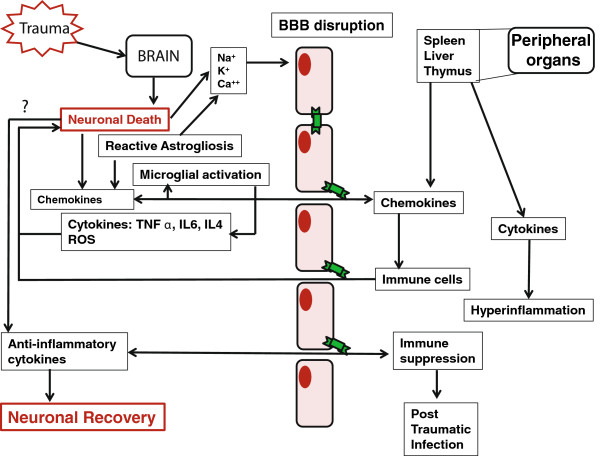
**Possible mechanism and the interactions between brain and systemic immunity after traumatic brain injury** (**TBI).** Blood–brain barrier (BBB) disruption allows peripheral immune cell infiltration into the brain. Interaction between brain and peripheral immune organs can cause either hyperinflammation or immune suppression. Anti-inflammatory cytokines may eventually lead to neuronal recovery.

Once the brain suffers mechanical insult, the injury process evolves over time and includes (a) primary injury caused by direct or indirect contusion resulting in shearing or stretching of brain tissue, subdural hematoma and cerebral ischemia (b) secondary injury characterized by diffuse axonal injury and inflammatory reactions, and (c) regeneration. The secondary, that is, the nonmechanical injury phase, is progressive and lasts from hours to days [13,14], significantly contributing to neurological disabilities [15]. Injury to the cerebral vasculature breaks the blood–brain barrier (BBB), allows entry of immune cells and stimulates inflammatory reactions. The molecular events result in apoptosis, inflammation, altered plasticity and neuronal regeneration. The complex nature of acute and chronic inflammatory reactions may aggravate the pathologic outcome or promote the repair process [16,17]. Also, multiorgan damage in trauma patients can lead to elevated circulatory levels of inflammatory cytokines that may contribute to the post-TBI pathogenesis of the brain [18] and cause multiple organ dysfunction syndrome (MODS) and death [19]. In this review we discuss the mechanism of interaction between the systemic immune response and the brain after TBI and current novel treatment approaches to combat TBI-induced damage (Figure 1).

### Response of the central nervous system to TBI: neuroinflammation and pathobiology of the CNS

The BBB protects the brain and maintains the homeostasis. Following TBI, a massive release of excitatory amino acid neurotransmitters, particularly glutamate, takes place [20,21]. These molecules interact with neurons and astrocytes and cause increased Ca^2+^, Na^+^, and K^+^ fluxes through overstimulation of glutamate receptors. As a consequence, catabolic processes are activated resulting in BBB breakdown [17]. The kinin system, excitotoxicity, activation of the innate immune system leading to neutrophil recruitment, mitochondrial alterations and microglial activation lead to generation of reactive oxygen species (ROS) which in turn trigger downstream pathways and cause oxidative damage, modifications in tight junctions and matrix metalloproteinase (MMP) activation. Thus ROS play an important role in mediating TBI-induced changes in BBB permeability [22]. ROS have also been implicated in fungal toxin T-2-mediated alteration in BBB permeability [23]. Recent animal studies have shown that BBB breakdown involves transcriptional changes in the neurovascular network and eventual neurodegeneration [24].

The leaky BBB allows the passage of inflammatory molecules and cells into and out of the injured brain initiating a cascade of responses in the brain and other organs. The most important events contributing toward the pathology of TBI are reactive astrogliosis, microglial activation, infiltration of immune cells in the CNS and neurodegeneration. Both the primary and secondary mechanisms of TBI cause neurodegeneration and contribute to post-traumatic neurological deficits [25,26]. One of the major pathological outcomes of these mechanisms is diffuse axonal injury (DAI), the main clinical feature of human TBI, leading to diffuse degeneration of cerebral white matter [27,28]. In a rodent model of diffuse TBI, Cernak *et al*. [26] have shown hypertension, brain edema, increased permeability of BBB, DAI and apoptosis of the cerebral cells following a high velocity impact. Alder *et al*. have characterized the pathological and behavioral changes in a lateral fluid percussion model (LFPI) of TBI in mice [29]. The process of TBI-induced neuronal cell death has multiple, overlapping and distinct molecular mechanisms [30]. Following TBI, neuronal cell death can be induced by caspase-dependent or -independent pathways [31], by cell cycle activation in which mature neuronal cells reenter the cell cycle and then die [32] or by autophagy [33]. In the caspase-dependent pathways, caspase 3 appears to play the major role in causing TBI-induced apoptosis, although caspase 6 and 7 have also been acknowledged as proapoptotic molecules [34]. The caspase-independent pathway is more complex and involves mitochondrial proapoptotic molecules including apoptosis-inducing factor (AIF) [35] and its regulators like PARP-1 [36,37], cyclophilin [38,39] and heat shock protein-70 (HSP-70) [40]. These mechanisms probably work together in stress-induced neuronal cell death and, therefore, inhibition of only one pathway may not be sufficient to protect neurons after TBI [41] (Table 1).

**Table 1:** Important inflammatory mediators in TBI

**Chemokines/ cytokines**	**Functions**	**Reference**
**CCL2**	Macrophage infiltration	Striling *et. al.* , 2004 [42]
**CCL20**	Inflammatory activator and immune cell attraction	Helmy *et. al.* , 2010 [43]; Comerford *et al*., 2010 [44]; Das *et. al.* , 2011 [45]
**CCL21**	Neuromodulatory	Biber *et. al.* , 2002 [46]; de Jong *et. al.* , 2005 [47]
**IL-1**	Neuronal injury	Rothwell, 1999 [48]
**IL-6**	BBB dysfunction, neuroprotection	Kossmann*et.al*, 1995 [49]; Penkowa *et.al.*, 2003 [50]
**IL10**	Neuroprotective	Kremlev and Palmer, 2005 [51]
**TNF-α**	BBB breakdown,	Kim *et. al.* , 1992 [52]
	Cerebral inflammation,	Ramilo *et. al.* , 1990 [53]
**IL-8**	Neutrophil infiltration	Whalen *et. al.* , 2000 [54]

### Role of neurocytokines and neurochemokines in the central response to TBI

In the 1980s, scientists observed that the brain, endocrine system and immune system function together to maintain homeostasis in health and prevent disease [55]. After Spangelo and co-workers identified cytokines and their role in inflammation and immunity [56], brain researchers began to study the actions of cytokines in the CNS. In 1992, Ban *et al.*[57] found that interleukin-1β (IL-1β) was synthesized in the brain under pathological conditions while others showed that peripherally synthesized cytokines were transported to the brain via the bloodstream or cerebrospinal fluid (CSF) and secreted into the brain parenchyma during breakdown of the BBB [58], thus linking the brain and immune system [59]. The chemokines are the chemotactic cytokines that play an important role in leukocytes migration [60]. Their role in signaling in the CNS was reported by investigators in the late 1990s [61-63]. Under inflammatory or neurodegenerative conditions in the CNS, chemokine molecules are synthesized by activated microglia or astrocytes which take part in the defense of the CNS by recruiting monocytes to the injury site [64-67]. Under normal physiological conditions the tight junctions of the BBB prevent infiltration of circulating leukocytes into the brain parenchyma [16,68]. Pathological conditions like infections, mechanical trauma or toxicity may disrupt the BBB and allow immune cells to enter the brain parenchyma in response to chemokine signaling from resident immune cells.

In addition to macrophages and glial cells, neurons have also been found to express chemokines and chemokine receptors in the brain under physiological and pathological conditions [2,62,69,70]. Fractalkine (CX3CL1) was the first chemokine seen to be constitutively expressed by the neuronal cells of the CNS [66]. Later, other chemokines like CXCL14/BRAK; [71,72], CCL20 [45], CCL21 [47], CXCL12/SDF-1 and CCL2/MCP-1, were found in neuronal cells under various pathological conditions including TBI. Helmy *et al.*[43] have reviewed the temporal profile of 42 cytokines after TBI in human patients. Upregulation of CCL20 has been observed in human subjects one day after severe TBI [43]. Furthermore, a recent study identified CCL20 as a dual-acting chemokine with the potential for inhibiting immune reactions and more importantly in attracting inflammatory effectors and activators [44]. Studies in our laboratory showed cerebral as well as systemic expression of CCL20 after mild TBI in rats [45]. Recently, Biber and co-workers [46,47,73] showed that damaged neurons produce CCL21, which assumes a neuromodulatory function. In a spinal cord injury model, Zhao *et al.*[74] have shown that CCL21 expressed by the damaged neurons used the CXCR3 receptor instead of the usual CCR7 receptor to activate the local microglial cells [75-77] and initiate inflammatory reactions. These neurochemokines can also be involved in nonimmune-related functions like neuromodulation or neurotransmission, which could be important in TBI. As Rostene and colleagues have pointed out, this could be the complex communication network between the neurons and the cells in its microenvironment that informs them about the damage [2].

In addition to chemokines, various cytokines have also been reported to be expressed following TBI, including TNF-α associated with activated microglia and astrocytes that may initiate the inflammatory process [78]. IL-6 in the injured brain has been associated with reactive astrogliosis, neuronal injury, and infiltration of peripheral cells [78-81]. TGF-β expression in the astrocytes and microglia after injury has been implicated in the pathology and dysfunction of the CNS and IL-1, IL-6, IL-8, IL-10, granulocyte colony-stimulating factor, TNF-α, FAS ligand and monocyte chemo-attractant protein 1 [18,82-84] are thought to account for the progressive injury. In a rat fluid percussion injury model a biphasic production of TGF-β, mainly of TGF-β 2, was detected in the ipsilateral cortex, with a first peak at 30 minutes and a second peak at 48 hours after the lesion. This response was accompanied by transient production of TNF-α and IL-6 occurring between five and eighteen hours after trauma. From this temporal pattern, Rimaniol *et al.* suggested an alternative pro- and anti-inflammatory role of TGF-β in the regulation of the brain cytokine network providing an endogenous mechanism for the control of the inflammatory reaction in traumatic brain injury [85].

### Activation of resident immune cells of the CNS following TBI

Microglial activation is integral to the response of the brain and spinal cord to injury [86]. A number of factors including pro-inflammatory and anti-inflammatory cytokines, chemokines, growth factors, nitric oxide, prostaglandins, and superoxide and other reactive oxygen species are released by microglia and modulate secondary injury as well as recovery after injury. Microglial activation is regulated in part by poly(ADP-ribose) polymerase-1 (PARP-1) [87]. Using a PARP-knockout mouse model of TBI, Whalen *et al.*[54] showed improved motor and cognitive functions after TBI and thereby indicated a detrimental role of PARP in the pathogenesis of TBI. In 2006, Bernardo and colleagues [88] observed that inhibition of microglial activation by peroxisome proliferator-activated receptor (PPAR)-gamma and its synthetic agonists by expression of surface antigens, synthesis of nitric oxide, prostaglandins, inflammatory cytokines and chemokines by TBI-induced brain inflammation could be controlled [88]. Perivascular macrophages are reactive cells that produce IL-1β and TNFα after CNS injury. In the perivascular endothelium these cytokines induce the expression of adhesion molecules and promote leukocyte infiltration [89].

### Response of the peripheral immune system to TBI: systemic immune activation and suppression after TBI

Multi-organ damage following TBI can lead to increased numbers of infiltrating inflammatory cells and levels of cytokines in the brain. Because of the compromised BBB, these cells and molecules gain access to the brain and aggravate the pathogenesis of TBI [18]. In spite of the importance of systemic inflammation and circulating inflammatory molecules in TBI, only limited investigations have been performed in this area. In a study on rats, Whalen *et al.*[54] observed systemic neutrophilia together with increased BBB permeability when granulocyte-colony stimulating factor (GCSF) was administered prior to cortical contusion injury (CCI). In another study Utagawa *et al.* demonstrated that systemically administered IL-1β markedly influenced the histopathological and behavioral outcome following fluid percussion injury. The leaking of pro-inflammatory molecules like cytokines, arachidonic acid metabolites, proteins of the contact-phase and coagulation systems, complement factors and acute-phase proteins, as well as hormonal mediators [90] through the compromised BBB into the circulation may generate a systemic immune response syndrome (SIRS) [90,91] characterized by hyper-inflammation or may release anti-inflammatory molecules targeting IL-1β, IL-6 or TNFα resulting in compensatory anti-inflammatory response syndrome (CARS) to block development of SIRS [19].

The production of inflammatory mediators is regulated by the negative feedback provided by the hypothalamus-pituitary-adrenal (HPA) axis and sympathetic nervous system (SNS) efferent limbs in CARS [19]; but in TBI, an imbalance between these two can lead to immunological dysfunction like organ damage or susceptibility to infections [91]. Stress-mediated release of cortisol and catecholamines can enhance the immune suppression. Direct infection through a skull fracture in TBI or from the transmigration of enteric bacteria after a closed head injury may cause infection, pneumonia and sepsis which can be life threatening in TBI or immune-compromised patients [10]. Griffin [10] has also pointed out that immune suppression after TBI causes retardation of healing in the brain infrastructure. In a 2001 human study, severe immune suppression was observed following severe TBI. Eighteen to seventy-two hours after head trauma, the numbers of circulating T-cells, T-helper cells, T-suppressor [92,93] and NK cells were reduced while the B-lymphocyte count remained normal [92]. There was also an increase in CD4+/CD45+ T cells [10,93]. The immune regulatory functions within the CNS following TBI, for example, microglia and astrocyte activation lead to antigen presentation to T-cells that alters their cytokine response and this may contribute to TBI pathology. On the other hand, the ability of these neuroantigen-reactive T cells to specifically infiltrate the CNS can be used to deliver molecules to augment a recovery response in degenerating CNS tissues [94].

### Response of peripheral immune organs to TBI

Despite ongoing research, the effect of TBI on other organs is largely unknown. In one study Mirzayan *et al*. [95] evaluated the histopathological changes in lung and liver. Following a single TBI event, they observed migration of immunocompetent cells to peripheral organs leading to various degrees of organ dysfunction. The spleen is a reservoir of peripheral macrophages and other immune cells in the body, and it is now well known that splenic signaling contributes to injury of various tissues after ischemic insult. For example, splenectomy prior to insult protects both the liver [96] and brain [84] from ischemic damage. They have also observed a reduction in spleen size following ischemic insult 84]. Li *et al.*[97] showed that splenectomy immediately after severe TBI induced by weight drop in rats decreased pro-inflammatory cytokine production, mortality rate and improved cognitive function. It was observed by Das *et al.*[45] that splenectomy immediately after the induction of mild TBI by lateral fluid percussion in rats attenuated neurodegeneration and CCL20 chemokine expression in the brain. Although the mechanism of spleen-brain interaction is not clear, it was found by Lee *et al*. [98] that the spleen participates in cerebral inflammation following intracerebral hemorrhage in a stroke model, as splenectomy reduced cerebral edema and inflammatory cell counts (probably by increased circulating catecholamines) [99]. Stewart and McKenzie [100] suggested that sympathetic stimulation can cause the release of immune cells from the spleen and subsequent infiltration into brain tissues. Regardless of the neural mechanism, removal of the spleen immediately after the insult would remove the largest pool of immune cells, which should decrease infiltration and consequent neuroinflammation. The thymus is the major source of maturing T-cells in the body. Although a great deal of investigation has been done to elucidate the relationship between brain trauma and the immune system, very little has been done to explore the function of the thymus after TBI. In a study of LFPI in rats, Das *et al*. found elevated CCL20 expression in the thymus following TBI [45]. Further investigation is needed to identify the specific function of thymus after TBI in adult rats. In a model of polytrauma combined with shock, Guan *et al.* observed apoptosis in the thymus, spleen, lung, liver and intestine which could cause the early organ injury and late organ failure seen in polytrauma patients [101]. In an effort to elucidate the hepatic response to acute brain injury, Campbell *et al.*[102] observed that clodronate-mediated Kupffer cell (KC) depletion reduced neutrophil- and ED-1-positive macrophage infiltration in IL-1β-injected brain or contusion-injured spinal cord by 70% and 50% respectively. Suppression of KC proliferation may, therefore, reduce secondary injury. Previously this group had pointed out that hepatic cytokines or chemokines produced as a result of acute injury may inhibit neutrophil recruitment to the CNS [102-105]. In recent studies, decreased liver weight and protein content, altered energy metabolism [106] and p450 dysfunction [107] have been observed following TBI.

### Cytokines and chemokines secreted peripherally control TBI

Following TBI, the signaling pathways are activated, inflammatory cells are mobilized and there is enhanced secretion of multiple inflammatory mediators like cytokines, chemokines and damage-associated molecular patterns (DAMPs). DAMPs in turn reactivate the inflammatory mediators and aggravate the damage [108]. The exact role of cytokines in brain trauma is not fully known, although experimental evidences suggest that cytokines play a major role in the body’s response to TBI. The major cytokines produced after TBI include tumor necrosis factor–alpha (TNF-α), IL-1β, IL-2, IL-6, IL-8, [91,109], IL-4 [110] and IL-18 [111]. Free radical nitric oxide (NO) is produced by the enzyme inducible NO synthase (iNOS) [112], which is an important inflammatory mediator after trauma in mice [113]. Among peripherally secreted chemokines in response to TBI the role of CCL20 has recently been described. This unique chemokine interacts specifically with the CC chemokine receptor 6 (CCR6) and induces chemotaxis of dendritic cells, T cells and B cells [114]. These cells are residents of the spleen and have the potential to promote neuroinflammation. CCL20 is expressed in inflamed epithelial cells [115] and in the synovial tissues of rheumatoid arthritis patients [116,117]. It has also been shown to be upregulated under normothermic conditions in a rat middle cerebral artery occlusion (MCAO) model [118]. Upregulation of CCL20 along with other cytokines has been observed in human subjects one day after severe traumatic brain injury [43]. Furthermore, CCL20 has been identified as a dual-acting chemokine with the potential for inhibiting immune reactions and more importantly in attracting inflammatory effectors and activators [44]. In a recent study using the LFPI rat model of TBI, Das *et al.* showed the expression of CCL20 mRNA and protein in spleen and thymus 24 hours after TBI, which is 24 hours before its expression in the brain. Since the thymus is the major source of mature circulating T cells, CCL20 expression in the thymus in adult rats as observed in this study seems significant [45] and should be further investigated.

### Is TBI associated with other neurodegenerative disorders?

There is increasing evidence showing that TBI is associated with neurodegenerative diseases like Alzheimer’s disease (AD), Parkinson’s disease (PD), multiple sclerosis (MS), and amyotropic lateral sclerosis (ALS) [119,120]. Epidemiological data indicates a single TBI event may trigger or accelerate the onset of Alzheimer’s disease (AD) in later life [121-124]. On the other hand, repetitive mild TBI has been associated with progressive neurodegeneration [125]. Since, Rudelli *et al.*[126] reported a case of classic AD pathology in a 38 year old severe head trauma patient, both tau pathologies and Aβ plaques were identified in survivors of single TBI [121,123] Subsequently, cases of AD-like pathology including neurofibrillary tangles and Aβ deposition [124,127-130] were reported in head trauma victims, including boxers, irrespective of age [131]. Although the Aβ plaques in AD and TBI are morphologically different, both contain primarily Aβ1- 42 with some occurrence of Aβ1-40 in TBI [129,130,132]. Aβ1-42 has also been observed in the CSF of severe TBI patients and is thought to be directly related to the increased level of cerebral Aβ [133] and neuronal amyloidogenic amyloid precursor protein (APP) levels after TBI [134]. Although results of animal studies on TBI induced AD pathologies are conflicting, it has been observed that post TBI activation of microglia and proinflammatory cytokine release exacerbates the AD like pathologies [135] in rats and is involved in APP processing that leads to generation of Aβ plaques [136,137].

In contrast to AD, studies attempting to correlate TBI and MS, another neurodegenerative, demyelinating disease of the CNS, are limited. Goldacre and colleagues [138] and Kurland [139] found no evidence of association between TBI and the development of MS. However, risk analysis using Taiwan’s National Health Insurance Research Database, indicated higher risk of incidence of MS in patients with a history of TBI compared to non TBI control group [140]. Parkinson’s disease (PD) is a neurodegenerative disorder, which affects the dopaminergic neurons of the substantia nigra. PD-associated mitochondrial dysfunction and pathology was observed after mild to moderate TBI and trichloroethylene (TCE) exposure in rats [141]. Also, TBI was reported to cause the nigrostriatal dopaminergic neurodegeneration in a rat model of LFPI suggesting that TBI is a risk factor of PD development [142]. Thus, although TBI appears to be associated with the development of some neurodegenerative diseases, conflicting data exist and detailed human and animal studies are necessary in this field. The most studied association between TBI and AD appears to suggest that TBI activation of immune mechanisms and proinflammatory cytokine activation of microglia contribute to neurodegenerative processes.

### Therapeutic approaches for TBI

A number of drugs for TBI have been tested in clinical trials but none has shown much promise. Most of the approaches to TBI therapy aim at treating the secondary neurodegeneration as a single component. Recently, a therapeutic regimen using multifunctional drugs has been proposed and tested in experimental neurotrauma models. The therapeutic agents included hormones like thyrotropin releasing hormone (TRH) and progesterone, heat shock proteins, neurotrophic factors, erythropoietin, statin drugs and antibiotics [143,144], substance P antagonists, cyclosporine, and magnesium salts among others [145].

### Anti-inflammatories for TBI

The inflammation following TBI causes tissue damage correlating with the secondary injury phase. Recently much attention has been drawn to the potential therapeutic benefits of inhibiting reactive oxygen species (ROS), reactive nitrogen species (RNS), and several types of tissue-digesting enzymes (matrix metalloproteinases), prostanoids, leukotrienes, and proinflammatory/inflammatory cytokines such as tumor necrosis factor-alpha (TNF-α). Inhibition of TNF-α with cannabinoids like pentoxifylline and dexanabinol, and use of corticosteroids or NSAIDs like ibuprofen or minocycline to reduce inflammation in the brain have shown promise in animals but failed in clinical trials [146]. Corticosteroids are a family of anti-inflammatory drugs that are widely used in autoimmune and allergic conditions and to reduce tumor-induced cerebral edema; but they failed to show any benefit in human trials of TBI involving adults and children [147]. Reduction of oligodendrocyte death and axonal degeneration by minocycline, a tetracycline derivative was observed in a spinal cord injury model [42]. Cederberg *et al*. [148] suggested that timing is crucial in inflammatory intervention, as IL-1, IL-6, and TNF-α may also play an anti-inflammatory role in a later stage of TBI-induced brain inflammation. Also, the PPAR-gamma agonist 15d-prostaglandin J(2) was shown to control brain inflammation by inhibiting microglial activation after TBI [88].

### Gene therapy for TBI

Gene therapy is a promising approach for the treatment of several diseases and conditions including TBI. With the advent of improved experimental techniques like microarrays for gene expression analysis, new targets are emerging for the treatment of diseases, drug development, immunotherapeutics and gene therapy. Colak *et al.* have identified several gene networks potentially involved in TBI that includes the C1ql2, Cbnl, Sdc1, Bdnf, MMP9, and Cd47 genes [149]. Redell *et al.* observed changes in hippocampal miRNA expression corresponding to the pathophysiological changes following injury and identified these as potential targets for gene therapy [150]. Degeorge and coworkers demonstrated that administration of viral-mediated glial cell-line derived neurotrophic factor (AdGDNF) one week prior to cortical contusion injury in rats resulted in neuroprotection but not functional recovery [151]. Attempts have been made to target mRNA translational regulation to combat neurodegeneration. Aberrant RNA oxidation, RNA degradation, altered RNA splicing and ribosomal changes – all leading to mRNA translational abnormalities have been described by many authors in different neurodegenerative conditions [152,153]. The mRNA translational regulation is affected by small non-coding microRNAs. The miRNA-argonaute complex suppresses the translation of target mRNA and each miRNA can regulate the translation of hundreds of mRNA targets and control the expression of many genes. Under cellular stress, a subset of microRNAs increases while expression of other miRNAs is decreased [154]. High throughput sequencing has shown that the human brain expresses over 1000 miRNAs, the functions of only approximately 500 of which have been determined [155]. MiRNAs have been implicated in various neurodegenerative conditions including TBI. Using microarray analysis, Redell and coworkers observed changes in the hippocampal expression levels of 444 miRNAs at 3 and 24 hours after controlled cortical impact injury in rats. In this study, 50 miRNAs were overexpressed including targets for proteins known to be initiated after injury [150]. Lei *et al*. also observed up- and down-regulation of rat cerebral miRNA up to 72 hours after TBI [156] while Liu *et al*. reported altered miRNA profiles after traumatic spinal cord injury in mice [157]. The potential exists for using miRNAs and small interfering RNAs (siRNAs) as therapeutic agents, but much work needs to be done before they will become a regular part of the physician’s tool kit. The si/miRNAs can be delivered using various transfection agents including liposomes, polyethylenimine (PEI), chitosan nanoparticles or by electroporation. Apart from the potential disadvantage of off-target effects, RNA knockdown can be useful in treating TBI.

### Transplantation-based approaches for treating TBI

In the past two decades, restorative therapeutic approaches focusing on repair or replacement of damaged or dead cells following TBI have gained importance [158]. Cellular transplantation is the method of choice because the brain itself has a limited capacity for self-repair. Early experiments with transplantation of fetal neural tissues with or without nerve growth factor (NGF) were effective [159], but raised issues of practicality and ethics. NT2N cells showed promise in graft survival [160,161]. It was found that *ex vivo* NGF gene therapy improved cognitive deficits following CCI in rodents [162,163]. Both rodent and human embryonic stem cells have shown encouraging results in survival, integration and attenuation of post-traumatic sequellae. Stem cells have the ability to self- renew and differentiate depending on specific cues. Neural stem cells in particular can divide unlimitedly and differentiate into neurons or glial cells. It was observed that E14.5 mouse embryonic stem cells transplanted with or without a fibronectin scaffold following CCI improved behavioral symptoms [164]. Xenotransplanted human neural stem cells have been found to survive in injured rodent brains and to express astrocytic and neuronal antigens [165,166]. They migrated to the hippocampus, corpus callosum and ipsilateral sub-ependymal zone [167] and decreased the number of degenerating neurons [168]. Bone marrow-derived stem cells (BMSCs), either hematopoietic or mesenchymal, are advantageous in that they can be harvested from the same animal and thereby avoid the problems of cell availability and immune rejection. These cells have successfully been transplanted into injured rats by different routes where they express neural and glial cell markers (35, 36) and migrate to the subventricular zone, hippocampus and pericontusional areas [169] indicating neurogenesis and improved neurobehavioral outcome [170]. Ma *et al.*[171] transplanted neural stem cells (NSCs) modified to encode brain derived neurotrophic factor (BDNF) in rats after TBI and found significant improvement in graft survival, neurogenesis and behavioral outcome. In another study in Wistar rats, functional improvement and colonization of BMSCs were observed after TBI and the recovery was found to be facilitated by granulocyte colony stimulating factor (G-CSF) [172]. Human fetal neural stem cells (hfNPCs) transplanted after CCI in SD rats increased angiogenesis and reduced astrogliosis [173]. As a long term effect they observed functional improvement, reduced lesion volume and increased neuronal survival surrounding the lesion [173].

The potential of therapeutic transplantation of immortalized progenitor cell lines after TBI, has also been tested by various authors. HiB5 cells derived from embryonic rat hippocampus [174,175], MHP36, the fibroblast growth factor 2 (FGF-2)-responsive Maudsley hippocampal cell line clone 36 [176] and C17.2, which is a clonal multipotent progenitor cell from murine cerebellum [177], have been tested for their efficacy in improving repair of the contusion site, migration, neurogenesis and neurobehavioral outcome. Hunang *et al.*[178] reviewed successful preclinical studies and clinical trials of cell-based therapeutics for different neurodegenerative conditions including TBI. They mentioned the use of restorative transplantation involving fetal/embryonic brain and spinal cord tissue, stem cells including embryonic, neural, hematopoietic, adipose-derived adult stem/precursor cells, skin-derived precursor and induced pluripotent stem cells, glial cells (Schwann cells, oligodendrocyte, olfactory ensheathing cells, astrocytes, microglia, tanycytes), neuronal cells (various phenotypic neurons and Purkinje cells), mesenchymal stromal cells originating from bone marrow, umbilical cord, and umbilical cord blood, epithelial cells derived from the layer of retina and amnion, menstrual blood-derived stem cells, Sertoli cells, and active macrophages. Functional recovery and angiogenesis were observed following transplantation of endothelial progenitor cells derived from adipose tissues in the injured rat brain [179] showing promise. Some of these approaches have also gone to clinical trials for SCI/TBI [180,181], and the clinical and scientific communities are paying more attention to the restorative treatment options for TBI.

## Conclusion

Traumatic brain injury is a complex process evoking systemic immune responses as well as direct local responses in the brain tissues. The primary or direct damage disrupts the BBB and injures the neurons. This initiates a cascade of inflammatory reactions including chemokine production and activation of resident immune cells. The leakage of the inflammatory molecules through the compromised BBB attracts peripheral immune cells to the site of injury. The effect of TBI is not restricted to the brain; it can cause multi-organ damage and evoke systemic immune response including cytokine and chemokine production. This facilitates the recruitment of immune cells to the site of injury and progression of the inflammatory reaction and subsequent repair processes. In spite of the socioeconomic burden of TBI and worldwide research efforts, an effective treatment is still not available. Translational regulation of mRNA by si/mi RNA shows promise as a safe and specific treatment to combat neurodegeneration. Transplantation-based therapies also have the potential to repair and restore brain structure and function but continued in-depth investigations are needed before they become successful therapeutics.

## Competing interests

The authors declare that they have no competing interests.

## Authors’ contributions

MD has researched and prepared the manuscript; SM and SSM have made critical suggestions on the content and reviewed the manuscript. All authors have read and approved the final manuscript.
